# The Complete Chloroplast Genome of a Medical Herb, *Myricaria squamosa* Desv. (Tamaricaceae), from Qinghai–Tibet Plateau in China

**DOI:** 10.3390/genes17080884

**Published:** 2026-07-29

**Authors:** Likuan Liu, Yifan Wang, Haoyu Liu, Tianyu Zhao, Jianfen Ma, Haotian Wang, Kaidi Su, Jinping Li

**Affiliations:** 1 Qinghai Provincial Key Laboratory of Medicinal Plant and Animal Resources of Qinghai-Tibet Plateau, School of Life Sciences, Qinghai Normal University, Xining 810008, China; 2025105@qhnu.edu.cn (L.L.); 15237886253@163.com (Y.W.);; 2 Plateau Science and Sustainability, Qinghai Normal University, Xining 810008, China

**Keywords:** *Myricaria squamosa* Desv., chloroplast genome, medical herb, Qinghai–Tibet Plateau

## Abstract

Background: *Myricaria squamosa* Desv., a perennial shrub of the Tamaricaceae family, holds significant ecological, medicinal and economic values on the Qinghai–Tibet Plateau. Methods: This study performed chloroplast whole-genome sequencing of *M. squamosa* and analyzed its genomic characteristics, codon usage bias, and phylogenetic relationships. Results: The *M. squamosa* chloroplast genome spans 154,689 bp, with a GC content of 36.3%. A total of 129 functional genes were annotated, including 84 protein-coding genes, 37 transport RNA (tRNA) genes, and eight ribosomal RNA (rRNA) genes. Structural analysis revealed that contraction and expansion of the inverted repeat (IR) boundaries in Tamaricaceae species primarily involve the *rps*19 and *ycf*1 genes. We identified 214 simple sequence repeat (SSR) loci. Comparative genomic analysis indicated higher variability in the large single-copy (LSC) and small single-copy (SSC) regions compared to the IR regions, and greater divergence in intergenic spacers than in protein-coding regions. All protein-coding sequences contain a total of 26,045 codons, with leucine (2750) being the most frequent, and a pronounced preference for adenine/thymine (A/T) endings. Phylogenetic reconstruction revealed that *M. squamosa* is most closely related to *Myricaria laxiflora*. Conclusions: This study provides valuable insights into the chloroplast genomic structure, codon usage patterns, and SSR distribution of *M. squamosa*, contributing to genomic evolution studies and taxonomic revision within the Tamaricaceae family.

## 1. Introduction

*Myricaria squamosa* Desv. (Tamaricaceae), is a fundamental component in traditional Tibetan medicine, where it is locally known as “Umbu”. The taxonomic classification of the genus *Myricaria* has long been a subject of debate, particularly concerning the taxonomic status of *Myrtama elegans*. The authors of [1,2] carried out a comprehensive taxonomic revision of the genus *Myricaria germanica* in China based on traditional morphological traits. They concluded that *M. germanica* is not present in China and proposed its geographical replacement as the newly described species *Myricaria paniculata*. Furthermore, they argued that certain morphological features of *Myrtama elegans*, such as the partial fusion of filaments at the base and the presence of white, long, soft hairs on the seed tips, are also observed in other species within the genus *Myricaria*, suggesting that *M. elegans* should not be classified under a separate genus. The morphological definition of species within the genus *Myricaria* has traditionally relied on type specimens and limited individual samples, which contributes to the ongoing challenges in achieving taxonomic consensus due to significant morphological differences and extensive interspecific hybridization [3]. Consequently, an increasing number of scholars worldwide are employing molecular biological techniques to address taxonomic uncertainties. Zhang et al. [4] collected samples representing 12 species from the genus *Myricaria*, as well as specimens from the genera *Tamarix* and *Reaumuria*. Amplification and sequencing of these samples were carried out using four molecular markers: ITS, *rps*16, *psb*B-*psb*H, and *trn*L*-trn*F. Phylogenetic analysis and molecular dating were conducted, employing the relaxed Bayesian molecular clock method implemented in BEAST. The results indicate that *Myricaria* comprises four distinct phylogenetic clades, each corresponding to a specific lineage within the genus. Molecular dating suggests that the initial diversification of *Myricaria* occurred during the early Miocene. Hu et al. [5] collected 25 samples from three species and 10 samples from other species within *Myricaria*, such as *M. laxiflora*. Phylogenetic tree analyses indicated that *Myricaria bracteata*, *M. squamosa*, and *M. paniculata* could not be clearly differentiated, forming two clustered monophyletic lineages collectively referred to as the *Squamosa* complex. Plastid phylogenetic analysis further revealed that this scaly *Myricaria* clade consists of two evolutionarily distinct units: one primarily distributed in the western Tarim Basin, and the other in the eastern Qinghai–Tibet Plateau. These findings contradict previous taxonomic classifications and suggest an urgent need for a re-evaluation and revision of the species boundaries within *Myricaria*. The desiccated shoots are extensively employed as medicinal remedies, exhibiting the therapeutic properties of dispersing exanthema, alleviating heat, and detoxifying. *Myricaria squamosa* exhibits a wide range of biological activities, including antibacterial, anti-inflammatory, analgesic, antioxidant, and anti-fatigue effects, as well as promoting cellular immunity, protecting against liver damage, and demonstrating anti-HIV properties [6].

The genus *Myricaria* comprises approximately 13 species, with a distribution centered on the Qinghai–Tibet Plateau and the northern temperate regions, and extending into Central Asia and Western Europe. In China, there are around 10 species and one variety [4,7]. They are predominantly found in mountainous areas, coastal regions, and sandy lands near lakes, at elevations ranging from 2400 to 4600 m, in provinces such as Qinghai, Tibet, Sichuan, Xinjiang, and Gansu. These species occupy critical ecological niches in wetlands, deserts, and riparian zones, where their robust root systems stabilize soils, mitigate erosion, and enhance water retention, underscoring their role in maintaining ecosystem resilience [8]. Chloroplasts possess their own genomes and serve as the primary sites of photosynthesis [9]. Their genomes exhibit high conservation in most higher plants, characterized by low mutation rates and gradual evolutionary dynamics, making them powerful tools for plant taxonomy and phylogenetics [10]. Advances in high-throughput sequencing have substantially reduced cost and turnaround time, enabling broad application of chloroplast genome data in species identification, phylogenetic reconstruction, and evolutionary studies. DNA barcoding, which uses one or more standardized short DNA regions to delimit taxonomic units, has gained accuracy, speed, and reliability through next-generation sequencing. Wang et al. [11] evaluated the efficacy of six candidate DNA barcode regions—ITS, ITS2, *psb*A-*trn*H, *mat*K, *rbc*L, and *trn*L-F—for species-level identification within the genus *Stephania*. The findings indicated that multi-locus barcode combinations achieved higher identification success compared to individual barcodes. Using both best match and best proximity criteria, the ITS+*psb*A-*trn*H combination exhibited the strongest discriminatory power, achieving a species identification rate of 93.93%. Shang et al. [12] identified four mutation hotspots (*acc*D-*psa*I, *atp*H-*atp*I, *ndh*C-*trn*V (UAC), and *ndh*F-*rpl*23) within the chloroplast genomes of seven *Ajuga* species, which can serve as valuable markers for distinguishing among species within this genus. Furthermore, chloroplast genome-based phylogenetic analyses have clarified the placement of Lysimachia within Primulaceae [13], establishing a framework to investigate interactions between the chloroplast genome, other plastid genes, and the nuclear genome. These findings underscore how integrating multi-locus barcodes and phylogenomic signals can improve the resolution and trustworthiness of species identification, while enhancing the interpretability of evolutionary relationships [14].

In this study, we sequenced and assembled the complete chloroplast genome of *M. squamosa*. The research foci of this study are as follows:

1. Characterize the complete chloroplast genome of *M. squamosa* through sequencing and assembly, elucidating its structural features, repeat sequences, and codon usage patterns.

2. Determine the evolutionary relationships and taxonomic position of *M. squamosa* by constructing a phylogenetic tree based on chloroplast genomic data.

3. Clarify phylogenetic demarcations within and across relevant genera by conducting comparative genomic analyses against related species.

Thus, we intend to furnish molecular genetic evidence that elucidates the evolutionary affinities of *Myricaria squamosa* chinensis and the phylogenetic demarcations within and across genera.

## 2. Materials and Methods

*Myricaria* plants were systematically collected from extensive regions across Qinghai Province and the northwestern part of Sichuan Province during the summers of 2022 and 2023.

### 2.1. Comprehensive DNA Extraction and Detection

After the complete grinding of the desiccated leaves, the total DNA was extracted utilizing the DP305 DNA extraction kit (TIANGEN BIOTECH, Beijing, China). For detailed operational procedures, kindly refer to the enclosed instructions provided with the kit. The concentration and purity of the extracted total DNA were determined using a micronucleic acid concentration tester (Thermo Fisher Scientific, Shanghai, China). The ratio of OD260 to OD280 for the highly pure DNA ranged between 1.7 and 1.9. A 2 μL aliquot of the DNA sample was mixed with 2 μL of 6× loading buffer, followed by agar gel electrophoresis at a voltage of 120 V.

### 2.2. The Assembly and Annotation of Chloroplast Genomes

The quality-assessed DNA samples were submitted to Novpgene Bioinformatics Technology Co., Ltd. (Beijing, China) for second-generation sequencing and paired-end sequencing on the Illumina HiSeq X platform (Illumina, Inc., San Diego, CA, USA). Raw reads were filtered using fastp v0.11.7 with the following criteria: reads containing adapter sequences were removed; reads in which >50% of bases exhibited a Phred quality score ≤ 5 were discarded; and reads with an N content exceeding 10% were eliminated. The resulting high-quality clean reads were used for plastid genome reconstruction [15]. The clean reads were compared with the previously published chloroplast genome of the *Tamarix* family, and subsequently, the reads associated with the chloroplast genome were chosen. These selected reads were then utilized for contig assembly using SOAPdenovo2 (r240) software, followed by subsequent optimization of the assembly outcomes [16]. The completeness and structural accuracy of the assembly were validated by mapping all clean reads back to the assembled genome; uniform coverage depth and concordant insert size distributions confirmed the absence of misassemblies and structural variants.

The complete chloroplast genome sequence of *M. squamosa* was annotated using Geneious Prime 2022.2.2 software, with manual adjustments made to the start codon and stop codon in the annotation results. Occasionally, the automatic annotation incorrectly assigned initiation codons (typically ATG) or termination codons (TAA, TAG, TGA) due to sequence noise or alignment biases, necessitating manual adjustments. For instance, the initial software annotation assigned non-standard start codons (e.g., GTG) to certain protein-coding genes, such as *rps*16 and *atp*F. These were subsequently corrected to ATG based on manual adjustments informed by homology-based comparisons with genes from closely related Tamaricaceae species, including *M. laxiflora* and *Tamarix ramosissima*. Annotation fidelity was further assessed by BLASTP searches against the NCBI non-redundant protein database (e-value < 1 × 10^−5^) and by verifying conserved domain architectures. Finally, the gene annotation and sequence information were uploaded to the NCBI site with registration number NC_060684. Using the Chloroplot online tools (https://irscope.shinyapps.io/Chloroplot/ (accessed on 24 April 2026)) [17], a chloroplast genome map was generated with a scale bar for *M. squamosa*.

### 2.3. The Analysis of IR Boundaries

In the study of chloroplast genomic structure evolution, the expansion and contraction of the IR region represent a key research focus. Variations in genome length among species are generally considered to be closely associated with changes in the IR region [18]. The GB files of nine species belonging to the *Tamarix* species were downloaded from the NCBI website, including *Tamarix taklamakanensis*, *T. ramosissima*, *Tamarix laxa*, *Tamarix karelinii*, *Tamarix arceuthoides*, *M. laxiflora*, *Myricaria prostrata*, *M. elegans* and *Myricaria Desv*. To analyze the contraction and expansion of IR boundary regions among these different species, we employed the online tool IRSCOPE available at https://irscope.shinyapps.io/irapp/ (accessed on 24 April 2026) [19].

### 2.4. The Analysis of Repeated Sequences

SSRs, also known as microsatellites, are short tandem repeats characterized by a high degree of uniformity in repeat unit length and base composition, with only minor variations observed. SSR molecular markers developed based on chloroplast genomes display significant genetic polymorphism both within and across species, making them widely applicable in the fields of population genetics and phylogenetic research [18]. The analysis of an SSR locus in the chloroplast genome of *M. squamosa* was conducted using the MISA-web online tool (https://webblast.ipk-gatersleben.de/misa/ (accessed on 24 April 2026)) [20]. The parameters were set as follows: the minimum number of repetitions for single nucleotides was set to ≥8, the minimum number of repetitions for dinucleotides and trinucleotides was set to ≥4, and the minimum number of repetitions for tetranucleotides up to six nucleotides was set to ≥3.

### 2.5. Comparative Analysis of the Genome

The chloroplast genomes of the *Tamarix* species, including *Reaumuria songarica*, *Tamarix arceuthoides*, *Tamarix chinensis*, *Tamarix gracilis*, and *Tamarix karelinii*, were converted from GB format to gb_mvista_annotation format using scaly *M. squamosa* as a reference. The visualization analysis of these chloroplast genomes was conducted using mVISTA online tools (https://genome.lbl.gov/vista/mvista/submit.shtml (accessed on 24 April 2026)) [21].

### 2.6. The Analysis of Codon Bias

The software Geneious Primer 2022.2.2 was utilized for exporting a total of 84 protein-coding sequences from the chloroplast genome of *M. squamosa*, while the CodonW 1.4.2 software was employed to analyze the codon usage frequency and relative codon usage frequency within the same chloroplast genome [22,23].

### 2.7. Phylogenetic Analysis

Complete chloroplast genome sequences of 16 species representing Tamaricaceae and Plumbaginaceae were retrieved in FASTA format from the NCBI database. The Tamaricaceae taxa comprised *Tamarix aphylla*, *Tamarix chinensis*, *Tamarix gracilis*, *T. ramosissima*, *T. laxa*, *Tamarix karelinii*, and *Tamarix arceuthoides*; *Myricaria wardii*, *Myricaria rosea*, *M. laxiflora*, *Myricaria prostrata*, and *M. elegans*; and *Reaumuria songarica* and *Reaumuria trigyna*. The outgroup consisted of *Plumbago zeylanica* and *Plumbago auriculata* (Plumbaginaceae). *M. squamosa* was included as the focal taxon.

Whole-chloroplast genome sequences were aligned using MAFFT v7 with the FFT-NS-2 algorithm under default parameters [24]. Phylogenetic trees were reconstructed using two independent methods: neighbor joining (NJ) and maximum likelihood (ML), both implemented in MEGA version 11 [25]. Branch support was assessed through bootstrap analysis with 1000 pseudoreplicates for both NJ and ML trees.

## 3. Results

### 3.1. Structural Features of the Chloroplast Genome

**Figure 1:**
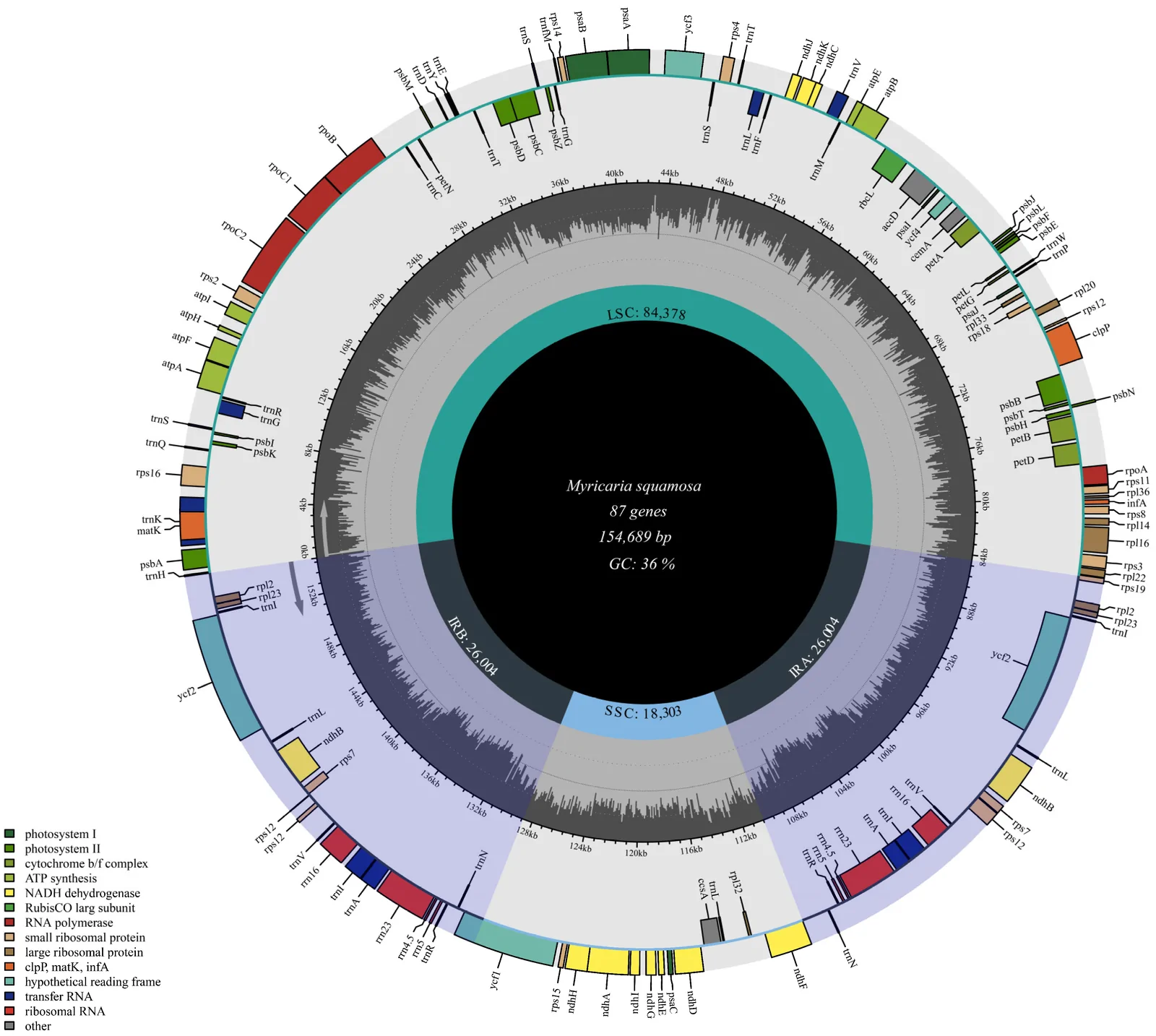
Gene map of *Myricaria squamosa* chloroplast genome. Genes are displayed on the interior and exterior of the circle. Different colors represent the functional categories of each gene. The black in the middle circle depicts the chloroplast structure, divided into four regions: the small single-copy region (SSC), large single-copy region (LSC), and two inverted repeats (IRa and IRb).

The chloroplast genome exhibited a total length of 154,689 base pairs (bps), featuring the characteristic four-segment circular double-stranded structure. Additionally, the GC content accounted for 36.3%. The LSC region has a length of 84,378 bp, the SSC region has a length of 18,303 bp, and the two inverted repeat (IR) regions have a combined length of 26,004 bp (Figure 1).

The chloroplast genome of *M. squamosa* harbors a total of 129 genes, comprising 84 protein-coding genes (PCG), 37 transfer RNA genes (tRNA), and eight ribosomal RNA genes (rRNAs). Among them, there were a total of 18 genes that exhibited copy gene characteristics (*ndh*B, *rps*7, *rps*12, *rpl*2, *rpl*23, *trn*V-GAC, *trn*R-ACG, *trn*N-GUU, *trn*L-UAA, *trn*L-CAA, *trn*I-GAU, *trn*I-CAU, *trn*A-UGC, *rrn*4.5, *rrn*5, *rrn*16, *rrn*23, *ycf2)*, all of which were precisely located within the reverse repeat region. There are 13 genes (*ndh*A, *ndh*B, *atp*F, *pet*B, *rpo*C1, *rps*16, *rpl*16, *trn*V-UAC, *trn*L-UAA, *trn*K-UUU, *trn*I-GAU, *trn*G-UCC *and trn*A-UGC) that each contain a single intron. Additionally, three genes (*rps*12, *clp*P *and ycf*3) possess two introns, as indicated in Table 1. Hu et al. [5] conducted a comparative analysis of the three species *M. bracteata*, *M. paniculata* and *M. squamosa*, which encoded a total of 88–90 PCGs and 8 rRNAs. Liu et al. [26] conducted an in-depth analysis of the complete chloroplast genome sequences of 12 *Tamarix* species, which includes 120 to 130 genes, encompassing 76 to 85 PCGs, 36 to 37 tRNAs, and eight rRNAs. This consistency confirms our gene count is within the typical range for the family.

### 3.2. IR Boundary Analysis

**Figure 2:**
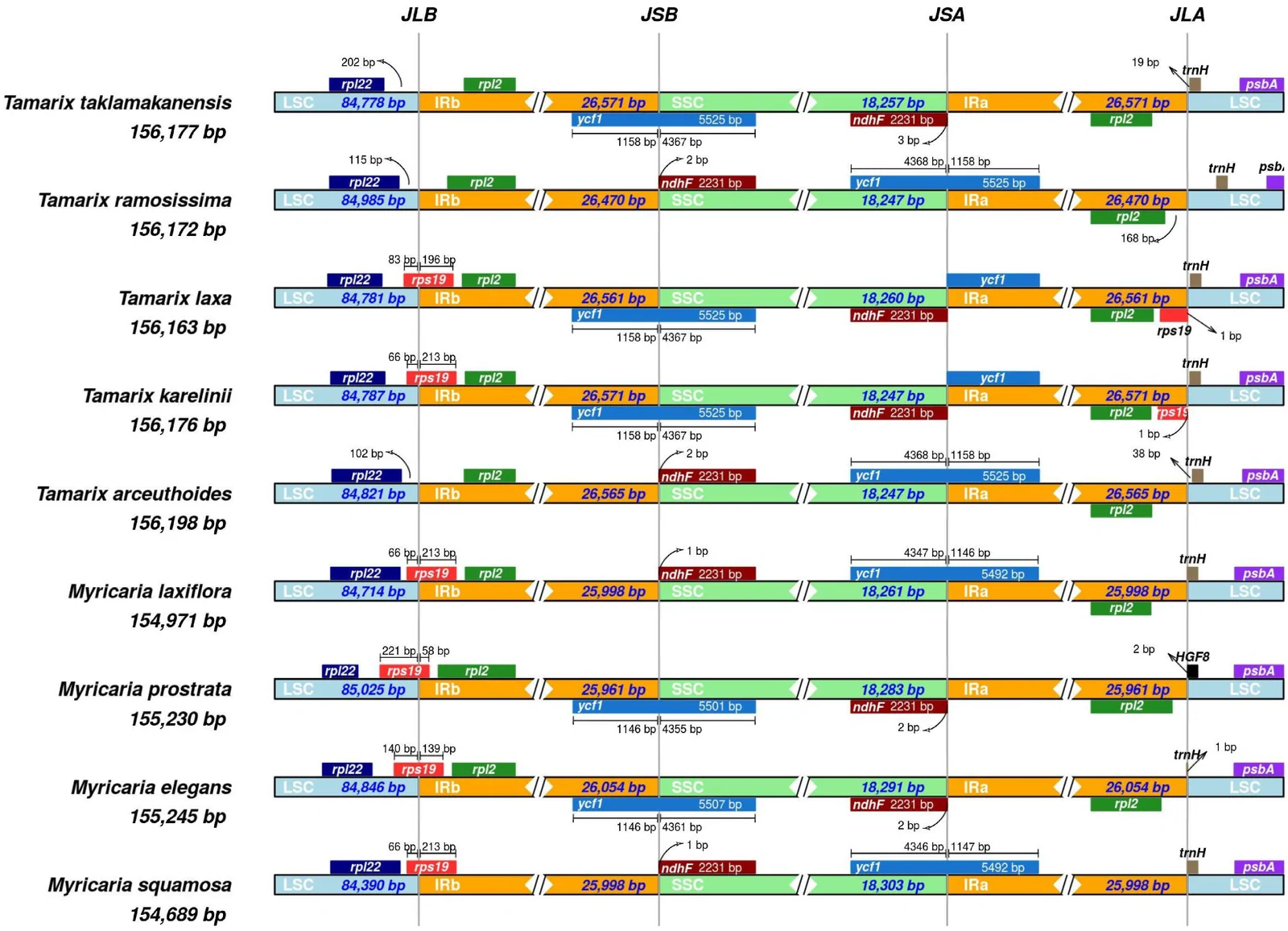
Schematic diagram of IR boundaries for 9 species in the Tamaricaceae family. Genes are represented by colored boxes, while arrows show the coordinate positions of each gene near the junctions. The gaps between the genes and boundaries are proportional to the distances in bps. Abbreviations denote the junction site of the plastid genome JLA (IRa/LSC), JLB (IRb/LSC), JSA (SSC/IRa), and JSB (IRb/SSC).

By comparing the chloroplast genomes of nine *Tamarix* species in the IR boundary region, it was observed that the LSC/IRb boundaries of four *Myricaria* species, *T. laxa*, and *Tamarix karelinii* were located on the *rps*19 gene. Conversely, the remaining three *Tamarix* species lacked the *rps19* gene. The boundaries between the inverted repeat B (IRb) and SSC regions of *T. taklamakanensis*, *T. laxa*, *Tamarix karelinii*, *Myricaria laxiflora*, and *Myricaria prostrata* sparsely flowering and creeping ramosarix were identified on the *ycf1* gene. Additionally, it was observed that a major portion of the *ycf1* gene is located within the SSC region. Furthermore, for other species, the IRb/SSC boundaries were found to be positioned 1–2 base pairs away from the *ndhF* gene. The *ycf*1 genes of *T. ramosissima*, *Tamarix arceuthoides*, *M. laxiflora*, and *M. squamosa* Desv were all found across the SSC/IRa boundary, with a majority of them located within the SSC region. The boundaries between *T. laxa* and *Tamarix karelinii* species were situated between the *ndh*F gene and *ycf*1 gene, while other species had boundaries 2–3 bp away from the *ndhF* gene. The IRa/LSC boundaries for all species were positioned at one end of the *trnH* gene or in close proximity to each other. Additionally, there was only a single base pair separating the *rps*19 gene from the TRNH gene in both *T. laxa* and *Tamarix karelinii* (Figure 2).

### 3.3. Repeat Sequence Analysis

The *M. squamosa* chloroplast genome contained a total of 214 SSR loci, comprising 151 mononucleotide repeats, 51 dinucleotide repeats, six trinucleotide repeats, and six tetranucleotide repeats. No pentanucleotide or hexanucleotide repeats were detected. The proportion of A/T in mononucleotide repeats was the highest, reaching 99.34%; in dinucleotide repeats, AT/AT accounted for the highest proportion, at 66.67%; similarly, in trinucleotide repeats, AAT/ATT had the highest proportion, at 66.67%; lastly, among tetranucleotide repeats, AATC/ATTG had the highest count and accounted for half of the total number (Table 2). The base composition of SSR loci in the chloroplast genome of *M. squamosa* is consistent with the high AT content (63.7%) observed across the entire genome.

### 3.4. Comparative Genomic Analysis

Based on the comparison results of the six selected species, it was observed that the degree of variation in the LSC and SSC regions exceeded that of the IR region, while the gene spacer region exhibited a greater degree of variation than the protein-coding region. The gene spacer regions, including *rps16-psbK*, *ndhC-atpB*, *PBM-PSBD*, *ndhF-rpl32*, and *rpl32-ccsA*, exhibited significant variations.

**Figure 3:**
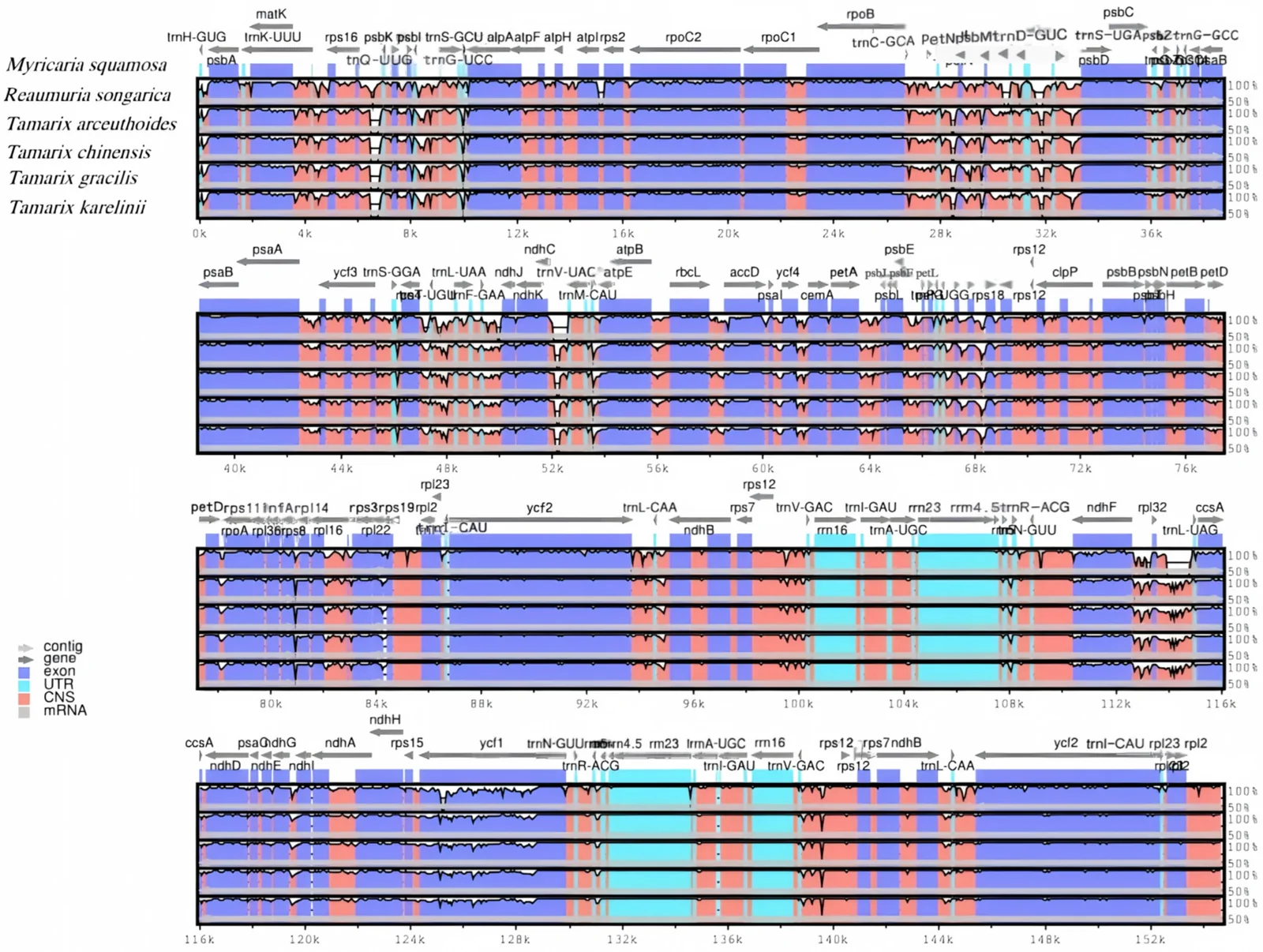
Comparative analysis of chloroplast genomes between *M. squamosa* and related species. The *y*-axis represents the percentage of interspecies consistency from 50% to 100%. Arrows represent genes and their directions. The dark blue area represents the protein-coding region, the cyan area represents rRNA or tRNA, and the orange area represents the gene spacer region.

The interspecific divergence between *Myricaria* and *Tamarix* was more pronounced in the *rps16-psbK* segment. The species difference between *Myricaria* and *Reaumuria* was more pronounced in *ndhC-atpB* and *rpl32-ccsA*. The *ycf1*, *accD*, and other genes exhibit simultaneous variations among different species (Figure 3).

### 3.5. Codon Usage Bias Analysis

After analyzing the protein-coding sequence in the *M. squamosa* chloroplast genome, it was observed that out of the 26,045 codons examined, leucine coding accounted for the highest proportion, with 2750 (10.56%), followed by isoleucine coding, with 2261 (8.68%). Conversely, cysteine codons were found to be least abundant, at 301 (1.16%) and TAA emerged as the preferred terminator codon, constituting approximately 58.8% of the total count. The relative synonymous codon usage analysis revealed that, out of the 30 codons with RSCU > 1, 13 and 15 had A or T as their terminal nucleotides, respectively. This observation indicates that the chloroplast genome of *M. squamosa* exhibits a preference for codons ending in A or T (Table 3).

### 3.6. Phylogenetic Relationships Analysis

**Figure 4:**
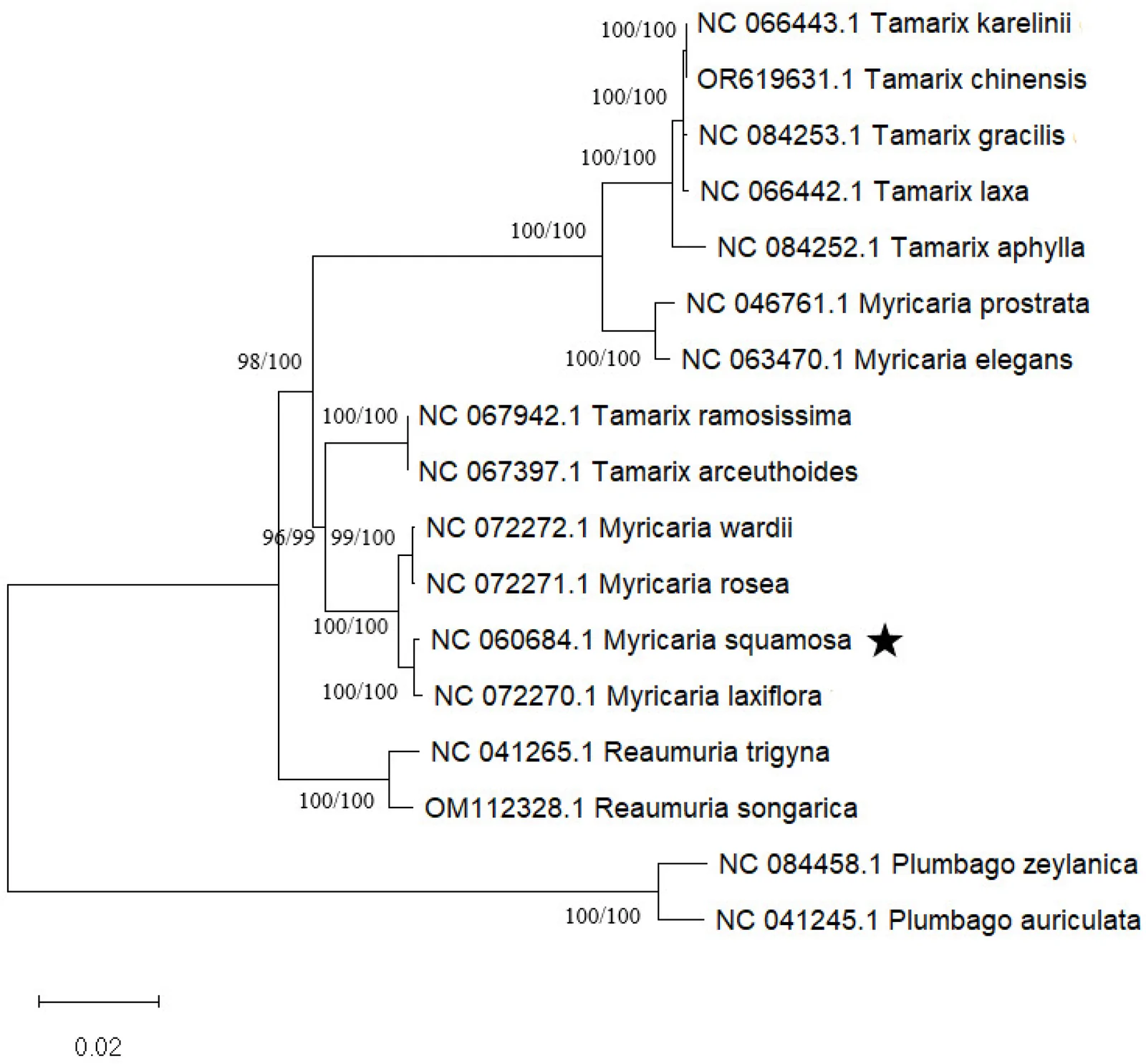
Chloroplast genome phylogenetic tree of 17 species, including *M. squamosa*, based on NJ and ML methods. Bootstrap values are shown near each node. ClustalW, integrated in MEGA-11 software, was utilized to perform sequence comparison, and phylogenetic trees were constructed using two methods: NJ and ML.

The phylogenetic trees reconstructed using neighbor joining (NJ) and maximum likelihood (ML) methods yielded identical topologies (Figure 4). Bootstrap support values were calculated from 1000 replicates to assess branch reliability. The two *Plumbago* species (Plumbaginaceae), designated as the outgroup, formed a distinct and strongly supported clade, confirming their distant relationship to Tamaricaceae. Within Tamaricaceae, the two *Reaumuria* species were resolved as a separate lineage, consistent with current taxonomic treatments. However, several topological features deviated from traditional generic delimitations. *Myricaria prostrata* and *M. elegans* grouped with five *Tamarix* species (*Tamarix karelinii*, *Tamarix chinensis*, *Tamarix gracilis*, *T. laxa*, and *Tamarix aphylla*) in one cluster. Moreover, *T. ramosissima* and *Tamarix arceuthoides* were placed with the remaining four *Myricaria* species in a second cluster, indicating that neither *Tamarix* nor *Myricaria* was recovered as monophyletic under the current sampling. Among all analyzed taxa, *M. laxiflora* was identified as the closest relative of *M. squamosa* with robust bootstrap support. These findings highlight phylogenetic incongruences that challenge the existing genus-level classification and underscore the need for an interpretable phylogenetic framework that can reliably resolve intergeneric boundaries within Tamaricaceae.

## 4. Discussion

The complete chloroplast genome of *M. squamosa* (154,689 bp, GC content 36.3%) exhibits the conserved quadripartite structure and gene content characteristics of Tamaricaceae [26], comprising 129 functional genes (84 protein-coding, 37 tRNA, and eight rRNA), 18 of which are duplicated in the inverted repeat regions. Thirteen genes contain one intron; *rps*12, *clp*P, and *ycf*3 contain two introns, a pattern conserved across Tamaricaceae. Retention of these introns suggests possible roles in RNA processing [27].

Inverted repeat boundary analysis across nine Tamaricaceae species revealed substantial structural variation with potential phylogenetic utility. The LSC/IRb boundary resides within rps19 in four Myricaria species, *T. laxa*, and Tamarix karelinii, whereas rps19 is absent from this junction in three other Tamarix species. The IRb/SSC boundary lies within ycf1 in *T. taklamakanensis*, *Tamarix laxa*, Tamarix karelinii, and two Myricaria species, but is positioned 1–2 bp from ndhF in the remaining taxa. These boundary shifts are not randomly distributed, but appear to coincide with major phylogenetic splits, suggesting they may serve as molecular markers for species delimitation [28]. However, the evolutionary mechanisms driving IR expansion and contraction—whether mediated by recombination, replication slippage, or selection—remain to be determined. Functional characterization of these boundary dynamics, particularly their impacts on gene expression or genome stability, would strengthen the interpretability of these markers beyond their descriptive value.

A total of 214 SSRs were identified. Mononucleotide repeats predominate (70.6%), with a strong A/T bias (99.34%), reflecting the genome’s AT-rich composition (63.7%). No pentanucleotide or hexanucleotide repeats were found. Among dinucleotide repeats, AT/AT is most abundant (66.67%), followed by AG/CT (33.33%), similar to *Tamarix chinensis*, but differing from *Reaumuria songarica*. These SSRs are candidate markers for population genetic studies. However, empirical validation is required before they can be used as ready-to-use markers [29].

Comparative analysis confirmed that the LSC and SSC regions exhibit substantially higher sequence divergence than the IR regions, and intergenic spacers are more variable than coding sequences, consistent with established patterns across angiosperms [30]. Five hypervariable regions were identified: *rps*16-*psb*K, *ndh*C-*atp*B, *psb*M-*psb*D, *ndh*F-*rpl*32, and *rpl*32-*ccs*A. Of these, *rps*16-*psb*K showed the greatest divergence between *Myricaria* and *Tamarix*, whereas *ndh*C-*atp*B and *rpl*32-*ccs*A were most variable between *Myricaria* and *Reaumuria*. The elevated variations in *ycf*1 and *acc*D further support their established utility as phylogenetic markers at lower taxonomic levels [30]. These regions collectively provide a robust toolkit for species-level discrimination within Tamaricaceae. However, functional annotation of variation in *acc*D, which encodes a subunit of acetyl-CoA carboxylase, is lacking; linking sequence divergence in this gene to possible adaptive significance would transform it from a purely diagnostic marker to a functionally informative locus.

Leucine is the most frequent amino acid (10.56%) and cysteine the least (1.16%). Among 30 codons with RSCU > 1, 13 end with A and 15 with T, reflecting the AT-rich genome composition, which is consistent with observations made in other plants [31]. The stop codon TAA is preferred (58.8%). Distinguishing mutational bias from translational selection would require tRNA or expression data [32].

The identical Phylogenetic analyses by NJ and ML analyses reveal that neither *Myricaria* nor *Tamarix* is monophyletic, with *Myricaria prostrata* and *M. elegans* embedded among *Tamarix* species and *T. ramosissima* and *Tamarix arceuthoides* nested within *Myricaria*. These results corroborate previously reported taxonomic uncertainties in Tamaricaceae and demonstrate that the morphological characters traditionally used to delimit these genera are homoplastic, underscoring the need for a molecularly grounded revision [5,33]. Beyond cataloging incongruence, the observed intermingling demands mechanistic explanation. Two population-level processes—chloroplast capture via hybridization and incomplete lineage sorting of ancestral polymorphisms—are both plausible given the weak reproductive barriers and sympatric distributions in this family, yet they cannot be distinguished using chloroplast data alone [34]. More fundamentally, these discordant relationships may also arise from lineage-specific mutation dynamics of the organellar genome itself. As articulated in the rethinking of mutation hypotheses for plant organellar DNA, nucleotide composition biases, strand-specific substitution asymmetries, and IR-mediated gene conversion can generate homoplastic molecular patterns that mislead phylogenetic inference if not explicitly modeled [35]. The pronounced AT bias and variable IR boundaries observed in Tamaricaceae chloroplast genomes are consistent with such mutation-driven convergence, suggesting that some topological conflicts may reflect heterogeneous mutational pressures, rather than true genealogical history. This interpretation shifts the focus from simply reporting discordance to evaluating the evolutionary processes that shape organellar sequence evolution.

Several methodological considerations delimit the immediate applicability of our results. The plastid genome, as a single, uniparentally inherited locus, cannot resolve complex evolutionary scenarios involving hybridization or introgression. Furthermore, in silico marker discovery, while efficient, generates hypotheses that require empirical validation before translation into conservation or population genetic tools.

## Figures and Tables

**Table 1:** List of genes of *Myricaria squamosa* chloroplast genome.

Category of Genes	Group of Genes	Names of Genes
Photosynthesis genes	ATP synthase genes	*atp*A, *atp*B, *atp*E, *atp*F *, *atp*H, *atp*I
	NADH dehydrogenase genes	*ndh*A *, *ndh*B * (×2), *ndh*C, *ndh*D, *ndh*E, *ndh*F, *ndh*G, *ndh*G, *ndh*H, *ndh*I, *ndh*J, *ndh*K
	Cytochrome b/f complex genes	*pet*A, *pet*B *, *pet*G, *pet*L, *pet*N
	Photosystem I genes	*psa*A, *psa*B, *psa*C, *psa*I, *psa*J
	Photosystem II genes	*psb*A, *psb*B, *psb*C, *psb*D, *psb*E, *psb*F, *psb*H, *psb*I, *psb*J, *psb*K, *psb*L, *psb*M, *psb*N, *psb*T, *psb*Z
Self-replication genes	RubisCO large subunit gene	*rbc*L
	RNA polymerase genes	*rpo*A, *rpo*B, *rpo*C1 *, *rpo*C2
	Ribosomal proteins (SSU) genes	*rps*2, *rps*3, *rps*4, *rps*7 (×2), *rps*8, *rps*11, *rps*12 ** (×2), *rps*14, *rps*15, *rps*16 *, *rps*18, *rps*19
	Ribosomal proteins (LSU) genes	*rpl*2 (×2), *rpl*14, *rpl*16 *, *rpl*20, *rpl*22, *rpl*23 (×2), *rpl*32, *rpl*33, *rpl*36
	Transfer RNA genes	*trn*Y-GUA, *trn*V-GAC (×2), *trn*V-UAC *, *trn*W-CCA, *trn*T-UGU, *trn*T-GGU, *trn*S-UGA, *trn*S-GGA, *trn*S-GCU, *trn*R-UCU, *trn*R-ACG (×2), *trn*Q-UUG, *trn*P-UGG, *trn*N-GUU (×2), *trn*M-CAU, *trn*L-UAG, *trn*L-UAA * (×2), *trn*L-CAA (×2), *trn*K-UUU *, *trn*I-GAU * (×2), *trn*I-CAU (×2), *trn*H-GUG, *trn*G-UCC *, *trn*G-GCC, *trn*fM-CAU, *trn*F-GAA, *trn*E-UUC, *trn*D-GUC, *trn*C-GCA, *trn*A-UGC * (×2)
	Ribosomal RNA genes	*rrn*4.5 (×2), *rrn*5 (×2), *rrn*16 (×2), *rrn*23 (×2)
Other genes	Protease gene	*clp*P **
	Maturase K gene	*mat*K
	Envelop membrane protein gene	*cem*A
	Subunit of acetyl-CoA gene	*acc*D
	C-type cytochrome synthesis gene	*ccs*A
	Hypothetical chloroplast reading frames	*ycf*1, *ycf*2 (×2), *ycf*3 **, *ycf*4
	Translational initiation factor gene	*inf*A

×2 indicates that the gene has two copies; * indicates that the gene contains one intron, and ** indicates that the gene contains two introns.

**Table 2:** Type and number of SSR loci of *M. squamosa* chloroplast genome.

Type	Repeats	Number	Location of Repeats			Proportion
			LSC	SSC	IR	
mononucleotide						
A/T	8	64	42	12	10	99.34%
	9	42	22	8	12	
	10	24	19	5	0	
	11	8	7	1	0	
	12	2	2	0	0	
	13	5	5	0	0	
	14	3	3	0	0	
	16	1	1	0	0	
	17	1	1	0	0	
C/G	8	1	1	0	0	0.66%
dinucleotide						
AG/CT	4	16	4	0	12	33.33%
	5	1	1	0	0	
AT/TA	4	24	20	2	2	66.67%
	5	4	4	0	0	
	6	2	2	0	0	
	7	1	0	1	0	
	8	2	0	0	2	
	9	1	1	0	0	
trinucleotide						
AAG/CTT	4	2	0	0	2	33.33%
AAT/ATT	4	4	2	2	0	66.67%
tetranucleotide						
AAAG/CTTT	3	2	2	0	0	33.33%
AAAT/ATTT	3	1	1	0	0	16.67%
AATC/ATTG	3	3	0	1	2	50.00%

**Table 3:** Usage of CDS sequence codons in chloroplast genome of *M. squamosa*.

Amino Acid	Codon	Number	RSCU
Phenylalanine Phe	TTT	1023	1.34
	TTC	499	0.66
Leucine Leu	TTA	887	1.94
	TTG	540	1.18
	CTT	594	1.30
	CTC	186	0.41
	CTA	381	0.83
	CTG	162	0.35
Serine Ser	TCT	603	1.80
	TCC	302	0.90
	TCA	421	1.25
	TCG	171	0.51
	AGT	400	1.19
	AGC	116	0.35
Proline Pro	CCT	421	1.60
	CCC	186	0.71
	CCA	307	1.17
	CCG	138	0.52
Isoleucine Ile	ATT	1136	1.51
	ATC	414	0.55
	ATA	711	0.94
Methionine Met	ATG	623	1.00
Valine Val	GTT	518	1.51
	GTC	152	0.44
	GTA	525	1.53
	GTG	177	0.52
Threonine Thr	ACT	549	1.67
	ACC	223	0.68
	ACA	410	1.24
	ACG	136	0.41
Alanine Ala	GCT	609	1.78
	GCC	214	0.63
	GCA	428	1.25
	GCG	115	0.34
Tyrosine Tyr	TAT	794	1.64
	TAC	175	0.36
terminator TER	TAA	50	1.76
	TAG	19	0.67
	TGA	16	0.56
Cysteine Cys	TGT	238	1.58
	TGC	63	0.42
Tryptophan Trp	TGG	454	1.00
Histidine His	CAT	475	1.54
	CAC	143	0.46
Glutamine Gln	CAA	719	1.56
	CAG	200	0.44
Arginine Arg	CGT	367	1.39
	CGC	87	0.33
	CGA	365	1.38
	CGG	113	0.43
	AGA	465	1.76
	AGG	185	0.70
Asparagine Asn	AAT	1007	1.57
	AAC	278	0.43
Lysine Lys	AAA	1082	1.54
	AAG	326	0.46
Aspartate Asp	GAT	853	1.57
	GAC	231	0.43
Glutamate Glu	GAA	1046	1.54
	GAG	317	0.46
Glycine Gly	GGT	566	1.33
	GGC	150	0.35
	GGA	728	1.72
	GGG	253	0.60

## Data Availability

All relevant data generated or analyzed during this study are included in this published article.

## Abbreviations

The following abbreviations are used in this manuscript:

NCBI	National Center for Biotechnology Information
RSCU	Relative synonymous codon usage
LSC	Large Single-Copy
NJ	Neighbor Joining
ML	Maximum Likelihood
SSC	Small Single-Copy
SSR	Simple Sequence Repeat
